# Quincy Medical Society

**Published:** 1867-09

**Authors:** Addison Niles

**Affiliations:** Secretary


### QUINCY MEDICAL SOCIETY.

The first quarterly meeting of the Quincy Medical Society was held at the office of Dr. Zimmermann, of Quincy, August 13th, 1867, at 10 o'clock A.M.

A quorum being present, the meeting was organized by the election of Dr. L. H. Baker, Chairman, in the absence of the President, Dr. C. A. W. Zimmermann.

The Secretary read the proceedings of the last two meetings, which were approved.

Dr. John S. Lower then moved that the Constitution and By-Laws proposed at the meeting held the 18th day of June last be adopted by this Society. This motion being seconded, was put and carried by a unanimous vote.

The amended Constitution makes it necessary for every member, in order to receive the right of suffrage, to present his credentials to the Society to be approved by a unanimous vote.

The Secretary then proceeded to present the credentials of nine of the twenty-nine members who compose the Quincy Medical Society, thirteen of whom reside in Adams County. After due examination of their credentials they were severally approved and the right of suffrage in this Society conferred upon the holders of each by a unanimous vote. The following were the credentials presented, viz.:

1. A Degree of Doctor of Medicine conferred upon John S. Lower, of Quincy, by the Cincinnati College of Medicine and Surgery, February 19th, 1845.

2. A Degree of Doctor of Medicine conferred upon Leander H. Baker, of Quincy, by the Louisville Medical Institute, March 3d, 1842.

3. A Degree of Doctor of Medicine conferred upon Addison Niles, of Quincy, by the Geneva Medical College, August 15th, 1835.

4. The credentials of C. A. W. Zimmerman, of Quincy, showing that he received a Degree of Doctor of Medicine conferred by the University of Gottingen, March 10th, 1835.

5. A Degree of Doctor of Medicine conferred by the University of Wursburg, June 4th, 1863, upon William Zimmerman, of Quincy.

6. A Degree of Doctor of Medicine conferred upon Joseph H. Reynolds, of Payson, by the Bellevue Hospital Medical College, March 1st, 1864.

7. A Degree of Doctor of Medicine conferred upon William Sigsbee, of Ellington, by the Castleton Medical College, June 16th, 1852.

8. A license to practice physic and surgery conferred upon Weller D. Rood, of Fowler, by the Onondaga County Medical Society, June 14th, 1840.

9. A license to practice physic and surgery conferred upon Andrew J. Miller, of Stone Prairie, by the Quincy Medical Society, Nov. 14th, 1865.

The Secretary then read a fee bill which had been proposed by the advice and concurrence of several members of the Quincy Medical Society.

On motion of Dr. William Zimmermann, it was unanimously adopted, and a Committee appointed to revise the same and report at the next regular meeting.

The Chairman appointed the following Committee for that purpose: Dr. A. Niles, Dr. William Zimmermann, and Dr. E. D. Helms.

The Secretary called the attention of the Society to the resolutions which were adopted by the East River Association of New York, June 20th, 1867, and published in the *Medical Recorder*, Aug. 1st, 1867, as follows:

*Whereas*, The attention of this Society has been called to consider the propriety of taking action relative to the practice of druggists renewing prescriptions of physicians without their written order, thereby injuring very materially the pecuniary interests of the profession, without gaining any particular benefit to themselves; and

*Whereas*, In view of the graver and more important considerations, that the interest and lives of patients are in consequence endangered, we consider it a duty to guard to the utmost of our ability against the liability to mistakes, which should be prevented rather than deplored; be it, therefore,

*Resolved*, That we cordially invite the earnest coöperation of every druggist in this city, especially in our immediate districts, to further this laudable purpose; and be it further

*Resolved*, That we respectfully request that no druggist will renew the prescriptions of any physician connected with this Society, without due authority for each and every such renewal; further, we will regard as unworthy of patronage any druggist who fails to comply with the requirements of these resolutions.

*Resolved*, That a copy of these resolutions, with a blank card, be sent to each and every druggist in our districts, with a request that the card be returned within two weeks to the Secretary, signifying their intentions with reference to compliance with these resolutions.

On motion, the above resolutions were adopted by the Quincy Medical Society, and the Secretary requested to present a copy to every druggist in the city, for his approval or dissent.

The Secretary reported that he communicated to the Secretary of the Adams County Medical Society the resolution adopted by the Quincy Medical Society, at its last meeting, as follows:

*Whereas*, A resolution was adopted by the Adams County Medical Society, held May 14th, 1767, inviting the Illinois State Medical Society to hold its next annual meeting in this city, which invitation has been accepted by the State Society; therefore

*Resolved*, That we cordially approve the measure, and hold ourselves in readiness to coöperate with the Adams County Society in making the necessary arrangements. For this end the Quincy Medical Society authorizes the President to appoint a Committee to confer with the Adams County Medical Society on the subject, and likewise to consider any propositions which may be brought forward to harmonize and unite the medical faculty in this city and county.

In accordance with the above resolution, the President appointed the following Committee, viz.:

Dr. E. D. Helms, John S. Lower, and L. H. Baker, of this city.

On motion of Dr. E. D. Helms, Dr. C. A. W. Zimmermann was added to the Committee.

Up to this date no official answer has been received from this communication.

On motion, the Society adjourned.

ADDISON NILES, Secretary.

